# The Heat Shock Protein 70 Family of Chaperones Regulates All Phases of the Enterovirus A71 Life Cycle

**DOI:** 10.3389/fmicb.2020.01656

**Published:** 2020-07-14

**Authors:** Yu-Siang Su, Pei-Yu Hsieh, Jun-Syuan Li, Ying-Hsuan Pao, Chi-Ju Chen, Lih-Hwa Hwang

**Affiliations:** Institute of Microbiology and Immunology, National Yang-Ming University, Taipei, Taiwan

**Keywords:** Enterovirus A71, heat shock protein 70, chaperone, viral life cycle, chaperone inhibitor

## Abstract

Enterovirus A71 (EV-A71) is one of the major etiologic agents causing hand, foot, and mouth disease (HFMD) in children and occasionally causes severe neurological diseases or even death. EV-A71 replicates rapidly in host cells. For a successful infection, viruses produce large quantities of viral proteins in a short period, which requires cellular chaperone proteins for viral protein folding and viral particle assembly. In this study, we explored the roles of the heat shock protein 70 (HSP70) chaperone subnetwork in the EV-A71 life cycle. Our results revealed that EV-A71 exploits multiple HSP70s at each step of the viral life cycle, i.e., viral entry, translation, replication, assembly and release, and that each HSP70 typically functions in several stages of the life cycle. For example, the HSP70 isoforms HSPA1, HSPA8, and HSPA9 are required for viral entry and the translational steps of the infection. HSPA8 and HSPA9 may facilitate folding and stabilize viral proteins 3D and 2C, respectively, thus contributing to the formation of a replication complex. HSPA8 and HSPA9 also promote viral particle assembly, whereas HSPA1 and HSPA8 are involved in viral particle release. Because of the importance of various HSP70s at distinct steps of the viral life cycle, an allosteric inhibitor, JG40, which targets all HSP70s, significantly blocks EV-A71 infection. JG40 also blocks the replication of several other enteroviruses, such as coxsackievirus (CV) A16, CVB1, CVB3, and echovirus 11. Thus, targeting HSP70s may be a means of providing broad-spectrum antiviral therapy.

## Introduction

Enterovirus (EV)-A71 belongs to the genus *Enterovirus* within the family *Picornaviridae* and is transmitted mainly through the fecal-oral route. After primary replication in the gastrointestinal tract, the virus disseminates and infects other tissues and organs, including the skin, heart, and/or central nervous system. EV-A71 infection usually manifests as mild illness such as hand, foot, and mouth disease (HFMD) or herpangina. However, in some small children, EV-A71 infection can progress to severe neurological diseases such as aseptic meningitis, brain stem encephalitis or acute flaccid paralysis, which are often associated with high mortality (Huang et al., 1999). With the successful control of poliovirus, EV-A71 has become one of the most clinically significant etiologic agents of acute neurological diseases (Nolan et al., 2003). Although an inactivated vaccine against EV-A71 has been recently launched (Li et al., 2014; Zhu et al., 2014), the monovalent vaccine can protect only against the infection of some, but not all, EV-A71 strains and not against infection of other EVs, such as coxsackievirus A16 (CVA16) (Chong et al., 2015). The regular outbreak of HFMD by EV infection remains a threat to small children.

Enterovirus A71 is a non-enveloped virus that contains a positive, single-stranded RNA genome encoding a single large polyprotein. The viral life cycle begins with the interaction of viral particles with cell surface receptors, scavenger receptor class B member 2 (SCARB2, expressed in a variety of cell types) (Yamayoshi et al., 2009) or P-selectin glycoprotein ligand 1 (PSGL1, expressed mainly on leukocytes) (Nishimura et al., 2009). SCARB2 mediates viral entry in a clathrin- and dynamin 2-dependent manner (Hussain et al., 2011; Lin et al., 2012), by which viral particles are transported from the cell surface *via* clathrin-coated pits into early and late endosomes. Endosomal acidification provides a cue to the virus to initiate the uncoating process.

Upon uncoating, viral RNA (vRNA) is released into the cytoplasm and translated into a polyprotein. The vRNA also serves as the template for the replication of the viral genome. The synthesized polyprotein is further processed by viral 2A^pro^ and 3C^pro^ to generate structural proteins (VP0, VP1, and VP3) and non-structural proteins (2A, 2B, 2C, 3A, 3B, 3C, and 3D). Similar to proteins of other EVs, the translation of EV-A71 is mediated by the internal ribosome entry site (IRES) located at the 5′ untranslated region (UTR) of the viral genome, which requires some canonical translational factors and several IRES *trans*-acting factors (ITAFs) (reviewed in Lozano and Martinez-Salas, 2015). The IRES-mediated mechanism allows viruses to continue translation while host cap-dependent translation is suppressed during viral infection. vRNA replication is carried out by 3D^pol^, which uses 3B (also known as VPg) as a primer for the synthesis of both positive- and negative-strand RNA (reviewed in Paul and Wimmer, 2015). In addition to 3D^pol^, the replication complex contains several other viral proteins or their precursors (2BC, 2B, 2C, 3A, and 3CD) and host factors [poly(rC)-binding protein 2 (PCBP2), polyA-binding protein 1 (PABP1) and heterogeneous ribonucleoprotein C (hnRNPC), etc], which were found to be associated with virus-induced membranous vesicles (Ilnytska et al., 2013; van der Schaar et al., 2016). EV infection induces the formation of autophagosomes (Jackson et al., 2005; Wong et al., 2008; Huang et al., 2009), and autophagy-associated membrane scaffolds may serve as sites for vRNA replication (Wong et al., 2008; Belov et al., 2012).

At the final stage of infection, the newly synthesized viral genome is packaged to form viral particles. The P1 precursor protein is first cleaved by 3CD protease to yield capsid proteins VP0, VP1, and VP3. Once cleaved, these three structural proteins automatically form protomers that subsequently oligomerize to form pentameric particles. Twelve pentamers then condense around the replicating RNA to form a provirion. Concurrently with encapsidation, the packaged RNA triggers VP0 protein cleavage into VP2 and VP4, forming 160S infectious viral particles (Basavappa et al., 1994). Interestingly, thus far, despite long-term studies, no specific packaging signal has been found on EV genomes. However, encapsidation specificity was found to be conferred by protein-protein interactions between viral VP3 and 2C in the replication complex (Liu et al., 2010). EV-A71 is typically considered a cytolytic virus that employs cell lysis for viral particle release. Cell apoptosis induced by the viral proteins 2A, 2B, or 3C can ultimately lead to cell lysis (Ventoso et al., 1998; Cong et al., 2016; Li et al., 2017). However, several groups have found that some picornaviruses, including poliovirus (PV), coxsackievirus B3 (CVB3), hepatitis A virus (HAV), and EV-A71, can be released *via* membrane-bound vesicles in a non-lytic manner (Feng et al., 2013; Bird et al., 2014; Robinson et al., 2014; Chen et al., 2015; Too et al., 2016). Some studies have also demonstrated that EVs exploit the secretory autophagy pathway to exit cells non-lytically (Bird et al., 2014; Robinson et al., 2014; Chen et al., 2015). Two groups have recently found that EV-D68 (Corona et al., 2018) and CVB3 (Mohamud et al., 2018) are able to inhibit autophagic flux *via* cleavage of synaptosomal-associated protein 29 (SNAP29), a component of the N-ethylmaleimide-sensitive activating protein receptor (SNARE) complex, and cleavage of an adaptor/tethering protein, pleckstrin homology domain-containing protein family member 1 (PLEKHM1), both by viral 3C^pro^. Both the SNARE complex and PLEKHM1 protein are essential for autophagosome and lysosome fusion (Itakura et al., 2012; McEwan et al., 2015). Because of the cleavage of SNAP29 and PLEKHM, the viruses are not degraded by autophagolysosomes but redirect autophagosomes to merge with late endosomes to facilitate viral exocytosis (Corona et al., 2018; Mohamud et al., 2018).

A successful viral life cycle requires the production of large quantities of viral proteins, presenting a large challenge in terms of viral protein folding and assembly. The production of large amounts of viral proteins during viral replication often leads to cellular stress and the upregulated expression of heat shock proteins (HSPs), which are likely involved in the folding of most viral proteins. Indeed, most viruses coopt host chaperone proteins to regulate their replication. Proteins in the human HSP70 family are among the most conserved chaperone proteins, with molecular weights that range from 66 to 78 kDa. The human HSP70 family contains at least 14 proteins that differ from each other by expression level, subcellular localization and amino acid composition (Stricher et al., 2013; Radons, 2016). Some of the HSP70s are stress-inducible, e.g., HSPA1 (also known as Hsp72) and HSPA6, whereas some are constitutively expressed, e.g., HSPA5 (also known as GRP78, with most ER-resident), HSPA8 (also known as Hsc70) and HSPA9 (also known as GRP75, with most mitochondria-resident). HSP70 chaperones facilitate protein folding in an ATP-dependent manner. They first bind with low affinity to unfolded proteins *via* cochaperone HSP40 in ATP-bound form. Following this binding, HSP70s hydrolyze the ATP into ADP, transitioning it to a high-affinity, ADP-bound state. Through the action of nucleotide exchange factors (NEFs), ADP is replaced with ATP, and the HSP70 chaperones revert to an ATP-bound state and release the polypeptide substrate. HSP70 chaperones participate in various steps of the viral life cycle. For example, HSPA1 facilitates replication complex formation for use by hepatitis C virus (HCV) (Chen et al., 2010). HSPA5 serves as a coreceptor for Japanese encephalitis virus (JEV) (Nain et al., 2017) or CVA9 (Triantafilou et al., 2002). HSPA8, the most abundant protein in the cell, regulates viral entry (Zarate et al., 2003; Chuang et al., 2015) or replication (Taguwa et al., 2015) and has been found to be associated with lentiviral or rabies viral particles (Sagara and Kawai, 1992; Gurer et al., 2002). HSPA8 has been reported to be involved in EV-A71 IRES-mediated translation (Dong et al., 2018). However, the interplay between the HSP70 chaperone network and throughout the whole EV-A71 life cycle remains largely uncharacterized.

In this study, we explored the roles of HSP70 family proteins in the EV-A71 life cycle. By combining an allosteric HSP70 inhibitor JG40 and using a *HSP70* knockdown strategy, we demonstrated that multiple members of the HSP70 family chaperones, such as HSPA1, HSPA8, and HSPA9, are involved in various stages of the EV-A71 life cycle, including virus entry, translation, replication, assembly, and exit. We found that EV-A71 replication is highly susceptible to the inhibitor JG40, which targets all HSP70s. Importantly, HSP70 inhibitor also blocks the replication of many other picornaviruses, such as CVA, CVB and echoviruses, which is indicative of the importance of HSP70s in the replication of most picornaviruses. Thus, targeting HSP70s may lead to a broad spectrum of antiviral strategies.

## Materials and Methods

### Cells and the Virus

RD (human muscle rhabdomyosarcoma; ATCC No. CCL-136) and HEK293T cells were cultivated in Dulbecco’s modified Eagle’s medium (DMEM; Gibco) supplemented with 10% fetal bovine serum, 2 mM L-glutamine, 100 U/ml penicillin, 100 μg/ml streptomycin and 0.25 μg/ml amphotericin at 37°C with 5% CO_2_. The EV-A71 strain (4643/TW/1998), kindly provided by Dr. Shin-Ru Shih (Chang-Gung University, Taiwan), was propagated in the RD cells and used to infect cells at the indicated multiplicity of infection (MOI). The CVA16, CVB1, CVB3, and Echovirus 11 were kindly provided by Dr. Szu-Hao Kung (National Yang-Ming University, Taiwan). All virus experiments were carried out in a biosafety level 2 (BSL2) laboratory, following the guidelines of the Center of Environmental Protection and Safety and Health, National Yang-Ming University, Taiwan.

### Plasmid DNA

The full-length cDNA replicon of EV-A71, SK-EV006 (Nishimura et al., 2009), was kindly provided by Dr. Hiroyuki Shimizu, National Institute of Infectious Diseases, Japan. To construct a firefly luciferase (Luc)-containing EV-A71 replicon, R1, the P1 region of SK-EV006, was replaced by a firefly luciferase gene. In brief, a 5′-UTR fragment containing an *Apa*I sequence at the 5′ end and *Pac*I + *Sac*II sequences at the 5′ end and a fragment consisting of P2 and part of P3 (P2 + part of P3), which contained a *Sac*II sequence at the 5′ end and an *Xho*I sequence at the 5′ end, was amplified by PCR using SK-EV006 cDNA as a template. The 5′ end of the P2 + part of P3 fragment retained the nucleotide sequences coding for the last two amino acid residues of the P1 protein, thus allowing 3C^pro^ cleavage at the P1 and P2 junction, and the *Xho*I sequence was an original site located in the middle region of the P3 gene. The 5′-UTR and the P2 + part of P3 fragments were first ligated *via* the *Sac*II site, and the resulting fragment was used to replace the corresponding region in the SK-EV006 cDNA sequence between the *Apa*I and *Xho*I sites, yielding the SK-EV006/P2+P3 intermediate. A Luc gene, which contained a *Pac*I sequence and an *Xho*I sequence at the 5′ and 5′ ends, respectively, was amplified by PCR and then inserted between the *Pac*I and *Sac*II sites of the SK-EV006/P2+P3 intermediate described above, yielding the R1 cDNA replicon. The R1 3D^D330A^ mutant replicon, in which an essential catalytic site of the 3D polymerase, Asp330, was mutated to an Ala, was constructed by PCR-based site-directed mutagenesis (Makarova et al., 2000) using the R1 replicon as a template. The monocistronic reporter plasmid (IRES-Luc), which contains EV-A71 IRES at the 5′ end and drives the translation of the luciferase protein, was described previously (Su et al., 2018).

### Antibodies and Reagents

The antibodies used in this study included mouse anti-EV-A71 VP1 from Abnova, mouse anti-EV-A71 VP0/VP2 (MAB979, which cross-reacts with the CVA16 capsid protein), mouse anti-CVB1 (MAB9410, which cross-reacts with CVB3) and mouse anti-Echo 11 (MAB9670) from Millipore, rabbit anti-EV-A71 VP3 and anti-HSPA9 from GeneTex, mouse anti-HSPA8 from Abcam, mouse anti-β-actin from Sigma, mouse anti-cleaved poly (ADP-ribose) polymerase (PARP) and anti-light chain 3B (LC3B) from Cell Signaling, mouse control IgG from BD Pharmingen, goat anti-mouse (H+L) conjugated to Alexa Fluor Plus 488 from Invitrogen, sheep anti-mouse IgG conjugated to horse radish peroxidase (HRP) and donkey anti-rabbit IgG conjugated to HRP from GE Healthcare. The antisera against EV-A71 2C, 3C, and 3D proteins were made in our own laboratory using purified recombinant proteins produced in *Escherichia coli*. The HSP70 inhibitor JG40 was synthesized according to a published procedure (Li et al., 2013) and provided to us by the Medicinal Chemistry Core Facility, Taichung, Taiwan. The fluorescent dye, 4′,6′-diamidino-2-phenylindole (DAPI); TRIzol reagent; and MEGAscript were purchased from Invitrogen. Bafilomycin A1 was purchased from Invivogen and cycloheximide was obtained from Sigma.

### Lentivirus-Based Knockdown, RNA Transfection, and Luciferase Reporter Assay

For knockdown experiments, lentiviruses expressing control shRNA (shCtrl) or shRNAs targeting specific *HSP70* genes were purchased from the National RNAi Core Facility, Taiwan. RD cells were infected with lentivirus at an MOI of 5 in the presence of polybrene (8 μg/ml), followed by selection with puromycin (2.5 μg/ml) for 2 days, and the resulting cells were used for subsequent experiments.

To perform RNA transfection, the R1 or R1 3D^D330A^ replicon RNA and the IRES-Luc reporter RNA were first transcribed *in vitro* from the corresponding plasmid DNA templates using a MEGAscript^TM^ kit (Invitrogen); 1 μg of the RNA was transfected into RD cells using Lipofectamine 3000^TM^ (Invitrogen) following the manufacturer’s instructions. The cell lysates were harvested at the indicated time points for RT-qPCR analysis or luciferase activity assay.

For the luciferase reporter assay, the RD cells transfected with the replicon RNA or IRES-Luc reporter RNA were harvested 9 h after RNA transfection. The lysates from one-half the cells were used for the luciferase activity assay using a Bright-Glo^TM^ luciferase assay system (Promega) following the manufacturer’s instructions. The other half of the cells were used for RNA extraction, and the transfected RNA levels were determined by RT-qPCR to indicate the transfection efficiency. The luciferase activity was normalized against the transfected RNA levels to reflect the translational efficiency.

### Quantitative RT-PCR (RT-qPCR)

Total cellular RNA was extracted using TRIzol reagent. cDNA was synthesized with a random hexamer using HiScript I Reverse Transcriptase (BIONOVAS Biotechnology Co. Ltd.). One-twentieth of the volume of the cDNA product was subjected to qPCR with primers targeting the viral *3D* region (for measuring vRNA levels), individual *HSP70* genes (for determining the knockdown efficiency), or glyceraldehyde-3-phosphate dehydrogenase (*GAPDH*, the internal control). qPCR was performed using FAST SYBR^®^ Green Master Mix (Thermo Fischer Scientific) following the manufacturer’s instructions. The levels of vRNA or *HSP70* RNA were normalized against the level of *GAPDH*. All primer sequences are listed in Supplementary Table S1.

### Viral Titration

Extracellular viruses were harvested from the cultured supernatant of virus-infected cells before cell lysis. The supernatant was centrifuged at 5,700 × *g* for 5 min to remove cell debris. Total virus were prepared by combining extracellular viruses with cell-associated viruses that were obtained by freeze-thawing the cells twice in 50 μl of the cultured supernatant and then removing cell debris by centrifugation at 15,300 × *g* for 10 min at 4°C. Viral titers were determined using a 50% tissue culture infective dose (TCID_50_) assay with RD cells according to the Reed and Muench method (Reed and Muench, 1938).

### Viral Internalization Assays

To examine viral internalization by confocal fluorescence microscopy, RD cells cultured on coverslips were treated with DMSO or JG40 (5 μM) together with cycloheximide (100 μg/ml), to prevent viral translation, 1 h prior to EV-A71 infection. The cells were infected with EV-A71 at 300 TCID_50_/cell and cultured for another 1 h to allow viral entry. After washes with PBS, the cells were fixed with ice-cold absolute methanol for 5 min. The cells were washed again with PBS and then permeabilized with 0.2% Triton X-100 at room temperature for 20 min. After a PBS wash, the cells were incubated with blocking buffer (5% FBS and 0.75% BSA in PBS) at room temperature for 1 h and then stained with anti-VP0/VP2 antibody (1:500, Millipore) at room temperature for 2 h, followed by a secondary antibody, goat anti-mouse IgG conjugated to Alexa 488 (1:200) at room temperature for another 2 h. Nuclei were stained by DAPI (0.5 nM) before the cells were mounted onto coverslips for processing. The specimens were imaged using a ZEISS LSM700 confocal microscope.

### Detection of Infection by Other Enterovirus With Immunofluorescence Assays

RD cells cultured on coverslips were treated with DMSO or JG40 (5 μM) 1 h prior to viral infection, and the drug was retained in the medium throughout the experiment. The cells were infected with CVA16, CVB1, CVB3 or Echo11 virus at an MOI of 1. At 9 h post infection (h.p.i.), cells were washed with PBS and fixed with 4% paraformaldehyde for 30 min. After 3 PBS washes, the cells were permeabilized with 0.2% Triton X-100 for 10 min. The cells were washed again with PBS, incubated with blocking buffer (5% FBS and 0.75% BSA in PBS) at room temperature for 1 h, stained with anti-CVA16 (1:1000), anti-CVB1 or anti-CVB3 (1:100) or anti-Echo 11 antibody (1:100) at room temperature for 2 h, followed by Alexa 488-conjugated to goat anti-mouse IgG secondary antibody, at room temperature for another 2 h. The nuclei were stained by DAPI, and the images were viewed on a Nikon ECLIPSE 80i fluorescence microscope at 100× magnification.

### Cycloheximide Chase Experiment

RD cells were infected with EV-A71 at an MOI of 5. The medium was refreshed with that medium containing DMSO or JG40 (5 μM) 2 h.p.i. Cycloheximide (100 μg/ml) was added to the medium 9 h.p.i. The cells were washed with PBS and lysed with Laemmli sample buffer 1, 2, 4, and 6 h after the cycloheximide treatment. An equal volume of each sample was applied to an SDS-PAGE gel for immunoblot analysis using antibody against 2C, 3C, 3D, or β-actin. The band intensities at different time points were quantified for each group by ImageJ software, with the values taken 1 h after cycloheximide treatment arbitrarily set at 100%. The regression curves were plotted using GraphPad Prism software.

### Coimmunoprecipitation

Cells were lysed in immunoprecipitation (IP) buffer containing 50 mM Tris–HCl (pH 7.4), 100 mM NaCl, 0.2% NP-40, 10 mM NaF, and 1 mM EDTA. The lysates were cleared by centrifugation at 15,400 × *g* at 4°C for 20 min. To perform immunoprecipitation, 500 μg of the lysates were incubated overnight with a control IgG or the indicated antibodies at 4°C. Then, 10–20 μl of protein-G sepharose beads (GE Healthcare) was added, and the samples were incubated at 4°C for 2 h. The beads were washed four times with ice-cold IP buffer, and the protein samples were heated at 95°C for 5 min in Laemmli buffer and then subjected to immunoblot analysis.

### Statistical Analysis

The data were analyzed by a two-tailed Student’s *t*-test or one-way ANOVA followed by Dunnett’s *post hoc* test (GraphPad Prism Software). ^∗∗∗^ indicates *P* < 0.001, ^∗∗^ indicates *P* < 0.01 and ^∗^ indicates *P* < 0.05. The differences were considered significant at *P* values < 0.05.

## Results

### Multiple HSP70 Family Chaperones Are Involved in the EV-A71 Life Cycle

**FIGURE 1:**
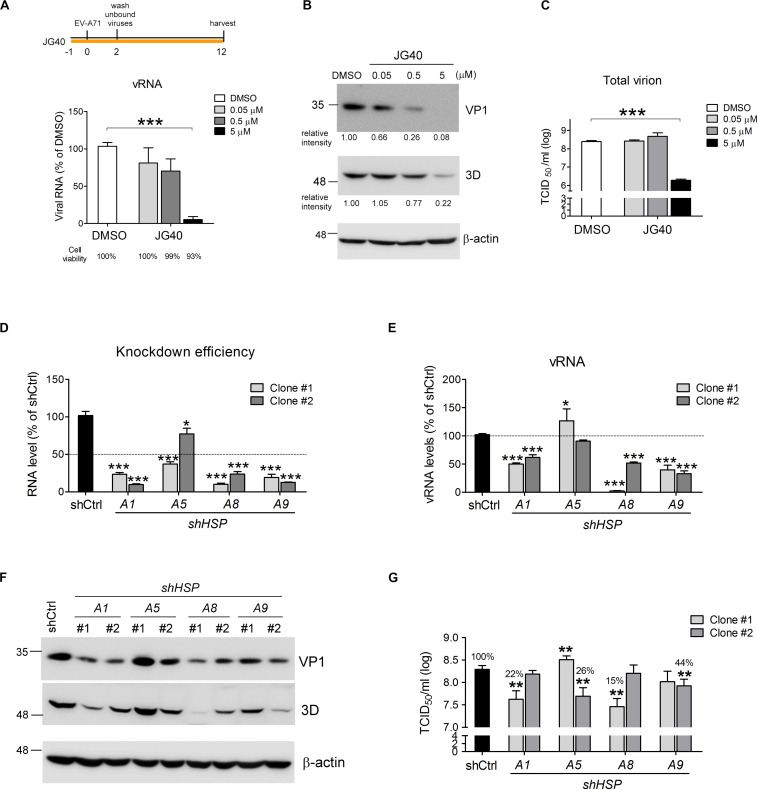
HSP70 family chaperones are required for the EV-A71 replication cycle. RD cells were treated with DMSO or the HSP70 inhibitor JG40 at the indicated concentrations 1 h prior to EV-A71 infection (MOI = 1). The drug was retained in the culture medium throughout the experiment. Twelve hours postinfection, **(A)** viral RNA was quantified by RT-qPCR using a primer pair targeting the 3D region. Cell viability, shown by the number below each bar, was determined by using alamarBlue methods following the manufacturer’s instructions; **(B)** the levels of the viral proteins VP1 and 3D were analyzed by immunoblot analysis using mouse antibodies. The relative expression levels are indicated by the numbers shown below the blot; and **(C)** total virus was prepared from the cultured supernatant and the cell lysates of the EV-A71-infected cells, and viral titers were measured by TCID_50_ assays. **(D)** Two independent shRNA clones targeting each of the *HSPA1*, *HSPA5*, *HSPA8*, or *HSPA9* genes were used separately to knock down the expression of individual HSP70s in RD cells. The knockdown efficiencies of all the shRNAs were measured by RT-qPCR using primers specific to the individual HSP70. The dotted line indicates 50% knockdown efficiency. **(E)** EV-A71 viral RNA replication levels in each of the *HSP70-*knockdown cells were measured by RT-qPCR 6 h.p.i. The dotted line indicates the vRNA levels in the control knockdown cells. **(F)** EV-A71 viral protein levels in the knockdown cells were analyzed 6 h.p.i. by immunoblotting using antibodies against VP1 and 3D. **(G)** Total viral titers were determined 12 h.p.i. by TCID_50_ assays. All the quantitative results are presented as the means ± standard deviation (SD) (*n* = 3), with the DMSO control **(A)** or the shCtrl-treated cells **(D,E,G)** set at 100%. One-way ANOVA followed by Dunnett’s *post hoc* analysis was performed to compare the differences between the DMSO group and the JG40-treated group **(A,C)** or between the shCtrl group and the knockdown groups **(D,E,G)**. *P < 0.05, **P < 0.01, and ***P < 0.001.

To explore whether HSP70 chaperones are involved in the EV-A71 replication cycle, an HSP70 inhibitor, JG40 (Supplementary Figure S1A, synthesized and kindly provided by the Medicinal Chemistry Core Facility, Taichung, Taiwan), was used to treat RD cells infected with EV-A71 at an MOI of 1 TCID_50_/cell. JG40 is an improved analog of MKT077 that blocks the interaction between HSP70 and NEFs, thus disrupting the turnover of ADP to ATP and impacting chaperone function (Li et al., 2013). Our experimental data obtained from 12 h post infection (h.p.i.) revealed that JG40 inhibited vRNA replication (Figure 1A) and viral protein synthesis (Figure 1B) in a dose-dependent manner. At 5 μM JG40, the titer of total virions, prepared by combining extracellular viruses with cell-associated viruses, was reduced by approximately 100-fold (Figure 1C). Notably, 93% of the virus-infected cells, relative to the cells in the DMSO-treated group, were still viable at this concentration (Figure 1A). Additionally, uninfected RD cells treated with JG40 at 0.05, 0.5 or 5 μM were mostly viable (98.3, 97.8, and 87.8% viability, respectively, Supplementary Figure S1B). These results indicated that the allosteric inhibitor JG40 induced little toxicity to host cells at the concentration that significantly inhibit EV-A71 replication. Together, these results indicate positive roles for HSP70 family chaperones in the EV-A71 life cycle.

The HSP70 family contains several isoforms. Therefore, we explored the roles of some ubiquitous isoforms, namely, HSPA1, HSPA5, HSPA8 and HSPA9, in the EV-A71 life cycle. Two separate shRNA clones specifically targeting each of these HSP70 isoforms were used to deplete individual HSP70s in the RD cells, and EV-A71 replication was examined in these knockdown cells. Most of these shRNAs had knockdown efficiencies greater than 50%, except for *shHSPA5* clone #2 (Figure 1D). In the cells depleted of HSPA1, HSPA8 or HSPA9, vRNA replication (Figure 1E) and viral protein synthesis (Figure 1F) were significantly reduced, thus reducing total viral titers down to 15–44%, respectively, compared to that of the shCtrl control (Figure 1G). Depletion of HSPA5 seemed to have minor effects on vRNA replication, with some conflicting results obtained on the knockdown efficiency, vRNA replication, viral protein synthesis and total viral titer. Thus, HSPA5 was not immediately pursued further in this study.

Notably, HSPA8 and HSPA9 showed 85% and 49% sequence identity to HSPA1 (Stricher et al., 2013). Therefore, the specificities of these shRNAs constituted a concern with respect to the knockdown experiments. To verify that these shRNAs would not downregulate the expression of other HSP70 isoforms in addition to isoform targeted, we concomitantly checked the RNA levels of the other two isoforms when one specific shRNA clone was used to knockdown a specific *HSP70* gene. The results showed that the two shRNA clones targeting *HSPA1* downregulated only *HSPA1* expression but not that of the other two *HSP70* isoforms (Supplementary Figure S1C), confirming their specificities and the important role of HSPA1 in the EV-A71 life cycle. The two shRNA clones targeting *HSPA8* not only downregulated *HSPA8* expression but also upregulated *HSPA1* expression 3–4-fold (Supplementary Figure S1D). Similarly, shRNA clone #2 targeting *HSPA9*, in addition to downregulating *HSPA9* expression, also upregulated *HSPA1* and *HSPA8* expression by approximately twofold (Supplementary Figure S1E). We reasoned that these phenomena could have been due to compensational effects, as both HSPA8 and HSPA9 are important constitutive chaperone proteins in the cell and depletion of them might lead to a cellular stress, which in turn would induce *HSPA1* expression. However, despite the elevated levels of HSPA1, depletion of HSPA8 or HSPA9 led to reduced EV-A71 vRNA replication and viral protein synthesis (Figures 1E,F), indicating their important roles in the EV-A71 life cycle. Taken together, these results indicate that the EV-A71 replication cycle requires HSPA1, HSPA8 and HSPA9.

### HSP70 Chaperone Proteins Are Required at All Stages of the EV-A71 Life Cycle

**FIGURE 2:**
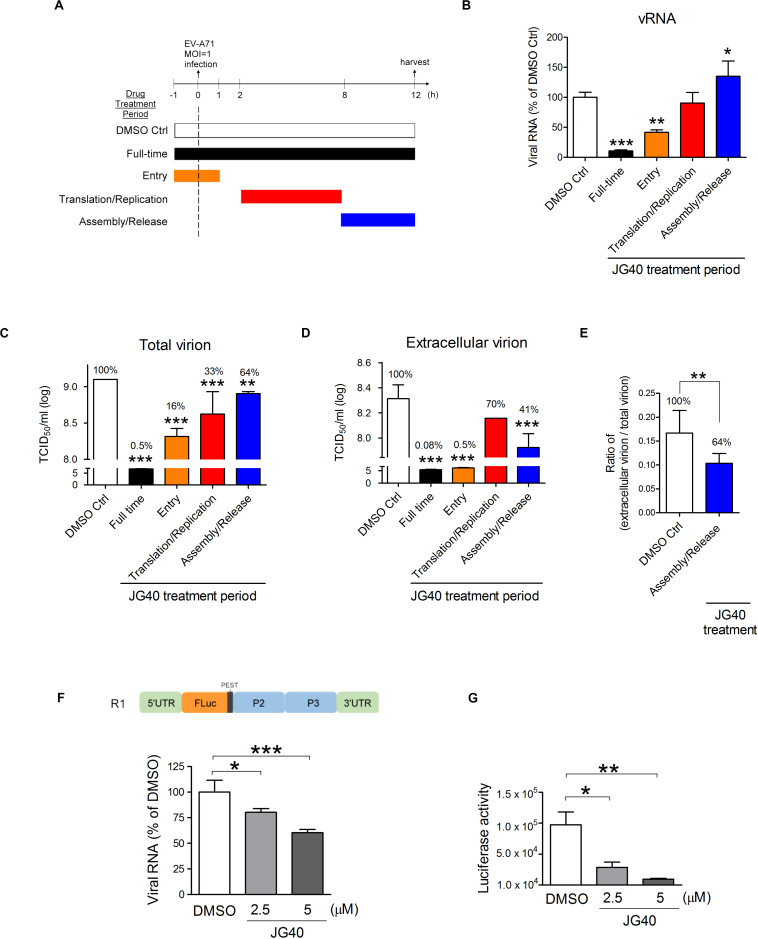
HSP70 family chaperones are involved in various stages ofthe EV-A71 life cycle. **(A)** Schematic diagrams show the timecourse of the time-of-addition experiments. RD cells were infected with EV-A71 (MOI = 1) at time 0 and harvested 12 h.p.i. for analysis. JG40 (5 μM) was either absent (DMSO Ctrl) or present throughout the experiment (full-time) or was present during a specific time period, e.g., –1∼1 h.p.i., 2∼8 h.p.i., or 8∼12 h.p.i., which roughly represented the times of viral entry, translation/replication and assembly/release stages, respectively. **(B)** Viral RNA levels were analyzed by RT-qPCR analysis based on the DMSO control and JG40-treated cells. **(C)** Total viral titers and **(D)** extracellular viral titers were determined by TCID_50_ assays, with the DMSO control set at 100%. **(E)** The ratio of extracellular viral titer/total viral titer, representing virion release efficiency, was determined with that of the DMSO-treated cells set at 100%. **(F)** The EV-A71 RNA replicon, R1 (shown in the schematic), was transcribed *in vitro*, and 1 μg of the RNA was introduced into RD cells by a transfection method in which the viral entry step is bypassed. RD cells were treated with DMSO or JG40 (2.5 or 5 μM) 1 h prior to RNA transfection and throughout the experiment. RNA was analyzed by RT-qPCR 3 h post transfection (h.p.t.) and **(G)** luciferase activity was analyzed 9 h.p.t. All the quantitative results are presented as the means ± SD (*n* = 3). One-way ANOVA followed by Dunnett’s *post hoc* analysis was performed to compare the differences between the DMSO group and the JG40-treated group. **(E)** Two-tailed Student’s *t*-test was performed to compare the differences between the ratios of the groups treated with DMSO or with JG40 at the assembly/release stage. *P < 0.05, **P < 0.01, and ***P < 0.001.

To understand which stages of the EV-A71 life cycle require HSP70s, we performed time-of-addition experiments. According to previous experiments performed in our laboratory, at an MOI = 1, approximately 13% of the infected cells would exhibit cytopathic effects (CPE) 12 h.p.i., with 30–40% of the cells lysing at 15 h.p.i. and > 92% of the cells dead within 24 h.p.i. Therefore, we administered JG40 to EV-A71-infected cells (MOI = 1) either throughout the whole experiment (full-time) or at three specific time periods, i.e., −1∼1 h.p.i., 2∼8 h.p.i. and 8∼12 h.p.i. which roughly represent the viral entry, translation/replication and assembly/release stages, respectively (Figure 2A). The cells were harvested 12 h.p.i. to determine the levels of vRNA and total viruses (cell-associated and extracellular). We also collected only extracellular viruses to determine the amount of virus released at this stage. The data revealed that, compared that of the DMSO control, cells treated with JG40 for the full period or at the entry stages showed markedly blocked vRNA synthesis (*P* < 0.001 and *P* < 0.01, respectively, Figure 2B) and total virus production (*P* < 0.001, Figure 2C) and extracellular virus production (*P* < 0.001, Figure 2D), indicating that HSP70s played important roles in EV-A71 entry into cells.

Administering JG40 at the translation/replication stage had no apparent effects on vRNA levels (Figure 2B) but significantly reduced total virus levels compared to the level in the DMSO control (*P* < 0.001, Figure 2C). Interestingly, administering JG40 at the assembly/release stage increased cell-associated vRNA levels (*P* < 0.05, Figure 2B) but reduced total virus and extracellular virus levels significantly (*P* < 0.01 and *P* < 0.001, as shown in Figures 2C,D, respectively). We reasoned that these conflicting results could be the result of blocked virus assembly and/or release caused by HSP70 inhibition; thus vRNA accumulated in the cells, but the total viral levels and outside viral levels were reduced. On the basis of the ratio of extracellular virions/total virions, which reflect the release efficiency, JG40 treatment of cells at the viral assembly/release stage was found to reduce this ratio to 64% that of the DMSO group (Figure 2E). Collectively, we conclude that HSP70s are also involved in EV-A71 viral assembly and release steps.

If JG40 blocked virus exit, then the conflicting data between the vRNA levels (Figure 2B) and viral titers (Figure 2C) for the cells treated with JG40 at the viral translation/replication stage could be explained by that HSP70s were also hypothesized to be involved in the translation/replication steps. According to this scenario, on the one hand, JG40 might reduce vRNA levels by inhibiting viral translation/replication but, on the other hand, promote the accumulation of vRNA in the cells by blocking virus release. It is highly likely that during the 2∼8 h.p.i. viral translation/replication and assembly/release concurrently progress to some extent. As a net result, the RNA levels were not significantly changed compared to those of the DMSO control, but the extracellular virus levels were reduced.

To verify that HSP70s participate in the viral translation/replication steps, RD cells were transfected with a Luc-expressing EV-A71 RNA replicon, R1, in which the P1 gene had been replaced with a firefly luciferase gene (Figure 2F), based on a method designed in which viral entry could be bypassed. Since the replicon lacks structural genes, it was not involved in the assembly/release processes. This approach allowed us to unequivocally demonstrate the role of HSP70s in the translation/replication steps. We first determined the translation and replication profiles of this replicon and found that vRNA replication peaked 3 h post transfection (h.p.t.) (Supplementary Figure S2A), and the luciferase activity peaked 9 h.p.t. (Supplementary Figure S2B). The results from this RNA replicon demonstrated that RNA replicon replication (Figure 2F) and luciferase activity (Figure 2G) were both inhibited by JG40 in a dose-dependent manner, indicating that HSP70s were indeed involved in EV-A71 translation/replication. Taken together, the time-of-addition results indicate that HSP70s are required at various stages of the EV-A71 life cycle, including viral entry, translation/replication and assembly/release.

### HSPA1, HSPA8, and HSPA9 Are Involved in EV-A71 Entry Into Cells

**FIGURE 3:**
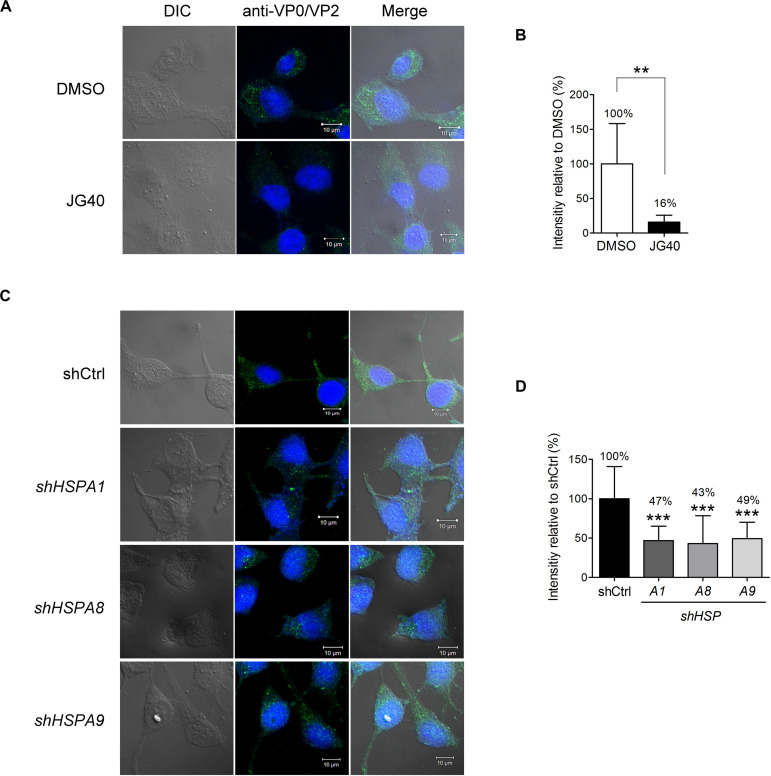
Multiple HSP70 chaperones are involved in EV-A71 entry into cells. **(A)** RD cells were treated with DMSO or JG40 (5 μM) together with CHX (100 μg/ml) 1 h prior to EV-A71 infection (MOI = 300). CHX was used to prevent viral RNA translation. After 1 h of infection, the cells were fixed and permeabilized. EV-A71 viral particles were detected with mouse anti-VP0/VP2 antibody, followed by Alexa Fluor 488-labeled secondary antibody. Cells were imaged by confocal fluorescence microscopy. (left) DIC image of the field; (middle) of stained viral VP0/VP2; and (right) the two images merged. One representative confocal slice is shown. Bar, 10 μm. **(B)** Quantitative comparison of the fluorescence intensity in the DMSO and JG40 groups is shown. The average integrated fluorescence intensity was calculated per cell based on the images taken of 10–15 fields (20–30 cells/field). The results are presented as the means ± SD of the integrated intensity of one representative experiment. Similar results were obtained from at least two independent experiments. **(C,D)** RD cells depleted of HSPA1, HSPA8 or HSPA9 and control knockdown cells were used for viral internalization experiments, and the integrated intensity was quantified as described in the legends to **(A,B)**, respectively. **(B)** Two-tailed Student’s *t*-test of the differences between the DMSO group and the JG40-treated group was performed. **(D)** One-way ANOVA followed by Dunnett’s *post hoc* analysis was performed to compare the differences in the shCtrl group and the knockdown groups. **P < 0.01, and***P < 0.001.

Next, we dissected the roles of different HSP70 isoforms in each step of the EV-A71 life cycle. To demonstrate the role of HSP70s in the EV-A71 entry step exclusively, JG40 and cycloheximide (CHX) were added to the cell culture 1 h prior to viral infection. The cells were infected with EV-A71 and incubated for 1 h to allow viral internalization and were harvested immediately for analysis. In the presence of CHX, vRNA translation was inhibited, which also prevented the subsequent replication step. After 1 h of viral infection, the cells were washed, fixed and stained with anti-VP0/VP2 antibody. The confocal microscopy images clearly showed significantly reduced fluorescence in the cells treated with JG40 (Figure 3A). A quantitative analysis revealed that the cells treated with JG40 had internalized only 16% of virus compared to that internalized by the DMSO control cells (Figure 3B). To examine which HSP70 was involved in the EV-A71 internalization step, RD cells depleted of individual HSP70s by one representative shRNA were infected with EV-A71 under conditions similar to those as described above. The results of the confocal fluorescence microscopy assay showed that depletion of HSPA1, HSPA8, or HSPA9 reduced virus internalization by the cells (Figure 3C), accounting for 47, 43, and 49% of the virus internalized, respectively, compared to the percentage internalized by the knockdown control cells (Figure 3D). Collectively, these data indicate that HSPA1, HSPA8 and HSPA9 are involved in EV-A71 entry into cells.

### HSPA1, HSPA8, and HSPA9 Regulate EV-A71 IRES Activity

**FIGURE 4:**
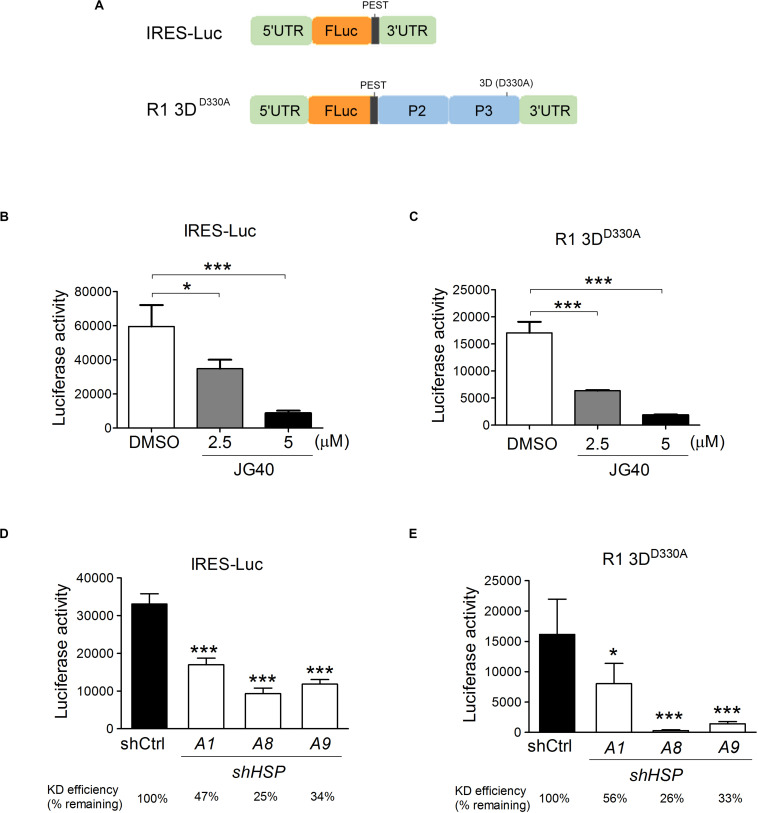
Multiple HSP70 chaperones are required for EV-A71 IRES translational activity. **(A)** Schematic diagrams show the monocistronic IRES-Luc reporter RNA and the R1 3D^D330A^ mutant replicon that had a D330A mutation in the 3D polymerase region of the R1 replicon. RD cells treated with DMSO or JG40 (2.5 or 5 μM) 1 h prior to RNA transfection and throughout the experiment were transfected with IRES-Luc RNA **(B)** or with the R1 3D^D330A^ replicon RNA **(C)**. IRES translational activity was determined by luciferase activity assays 9 h.p.t. To determine the IRES activity in the *HSP70-*knockdown cells, RD cells were first infected with lentiviruses expressing control shRNA (shCtrl) or shRNA targeting *HSPA1*, *HSPA8*, or *HSPA9*. The cells were then transfected with IRES-Luc RNA **(D)** or with R1 3D^D330A^ replicon RNA **(E)**. The knockdown efficiency of each shRNA in each transfection experiment is indicated at the bottom. IRES activity was determined by luciferase activity assays 9 h.p.t. One-way ANOVA followed by Dunnett’s *post hoc* analysis was performed to compare the differences between the DMSO group and the JG40-treated group **(B,C)** or between the shCtrl cells and the *HSP70*-knockdown cells **(D,E)**. *P < 0.05 and***P < 0.001.

To examine the roles of HSP70s in EV-A71 translational activity, two reporter RNAs were constructed (Figure 4A): The first was a monocistronic reporter RNA, IRES-Luc, for which the translation of luciferase was driven by EV-A71 IRES from the 5′-UTR of the viral genome; the second was a mutant viral replicon, R1 3D^D330A^, which had a D330A mutation in the 3D polymerase region of the R1 replicon and was replication deficient (Supplementary Figure S2A). The RNA replicon, however, still served as a template for luciferase protein translation *via* IRES activity for as long as 12 h post transfection (Supplementary Figure S2B). The luciferase activity based on these two reporter RNAs were true measures of the translational activity since no vRNA was replicated. This vRNA replicon expressing all the non-structural proteins was included here because, in our previous studies, we found that viral 2A^pro^ and 3C^pro^ significantly impacted IRES activity (Su et al., 2018); therefore, translation from this RNA replicon likely reflects natural viral IRES activity more closely. The luciferase from both constructs contains a PEST (Pro, Glu, Ser, and Thr) sequence at the carboxyl end, which confers a short half-life to the protein, allowing the measured luciferase activity to reflect the true translational activity and not merely the accumulated protein levels. We used a transfection method to directly deliver the *in vitro* transcribed reporter RNAs into RD cells that had been treated with different doses of JG40. The luciferase activity levels measured 9 h.p.t. in the cells with the IRES-Luc (Figure 4B) or R1 3D^D330A^ replicon (Figure 4C) revealed that JG40 blocked IRES activity significantly and in a dose-dependent manner. Reporter RNAs were also transfected into RD cells in which a specific HSP70 was deleted. Interestingly, HSPA1, HSPA8, and HSPA9 all seemed important for the translation of the IRES-Luc (Figure 4D) and 3D^D330A^ replicons (Figure 4E), especially HSPA8 and HSPA9, since depletion of either one markedly reduced the translational activity of the R1 3D^D330A^ RNA replicon (Figure 4E). Collectively, these results highlight the importance of HSP70 family chaperones in regulating EV-A71 IRES activity, particularly in the context of viral gene expression.

### HSPA8 and HSPA9 Facilitate the Formation of the EV-A71 Replication Complex by Stabilizing Viral Proteins

The translation and replication steps of EVs are tightly linked and may largely overlap in time. Hence, it is technically difficult to distinguish the replication period from the translation period *in vivo*. Given that most HSP70s are involved in EV-A71 translation, as illustrated above, and that translation is a prerequisite for viral replication, we were unable to decipher the roles of the HSP70s in the replication step *in vivo* by simply measuring vRNA or viral protein levels. However, we intuitively thought that HSP70s, as chaperones, might facilitate folding and thus enhance the stability of viral proteins involved in the replication process. Thus, we took an alternative approach to explore the roles of HSP70s in EV-A71 replication by examining the stability levels of the EV-A71 2C, 3C, and 3D proteins, known to be involved in the replication complex, in the presence or absence of JG40.

**FIGURE 5:**
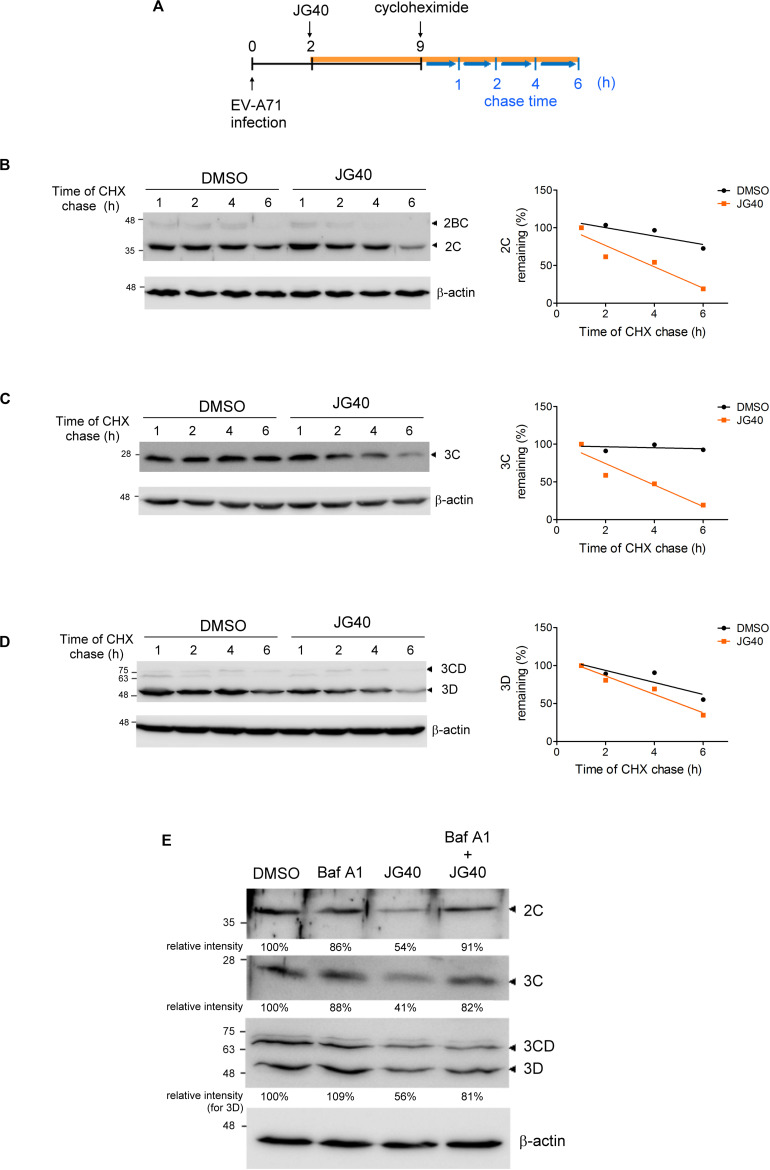
HSP70 inhibition decreases the stability of the EV-A71 2C, 3C, and 3D proteins in the EV-A71-infected cells. **(A)** Schematic diagram shows the time course of the CHX chase experiment during EV-A71 infection. RD cells were infected with EV-A71 (MOI = 5) at time 0. DMSO or JG40 (5 μM) was added to the cell culture 2 h.p.i., and CHX (100 μg/ml) was added 9 h.p.i. to prevent further protein synthesis. Viral protein levels remaining at 1, 2, 4, and 6 h after the CHX treatment were examined by immunoblot analysis. The intensities of the protein bands were quantified by ImageJ software, with the values 1 h after CHX treatment arbitrarily set at 100%. The stability levels of the EV-A71 **(B)** 2C, **(C)** 3C, and **(D)** 3D proteins in the DMSO-treated and JG40-treated cells were compared by the regression curves (GraphPad Prism), shown on the right. **(E)** EV-A71-infected RD cells (MOI = 5) were treated with bafilomycin A1 (100 nM) or JG40 (5 μM) alone or cotreated with JG40 and bafilomycin A1 2 h.p.i., and the levels of the EV-A71 2C, 3C, and 3D proteins were determined by immunoblotting 5 h.p.i. using specific antibodies. The relative intensities of the protein bands are shown at the bottom of each lane.

RD cells were treated with DMSO or JG40 at 2 h.p.i. to avoid drug effects on the viral entry step. CHX was added to the cell culture 9 h.p.i to prevent further protein synthesis, and the stability of the remaining protein was chased for another 6 h (Figure 5A). The results showed that the stability levels of the EV-A71 2C (Figure 5B), 3C (Figure 5C), and 3D (Figure 5D) decreased to some degree in the presence of JG40, indicative of the roles of HSP70s in stabilizing these viral proteins. The JG40-induced reduction in the 2C, 3C and 3D protein levels was significantly abrogated by bafilomycin A1, a lysosomal degradation inhibitor (Figure 5E), but not by MG132, a proteasomal degradation inhibitor (data not shown), implicating that the decreased stability of viral proteins upon HSP70 inhibition was mediated through lysosomal degradation.

**FIGURE 6:**
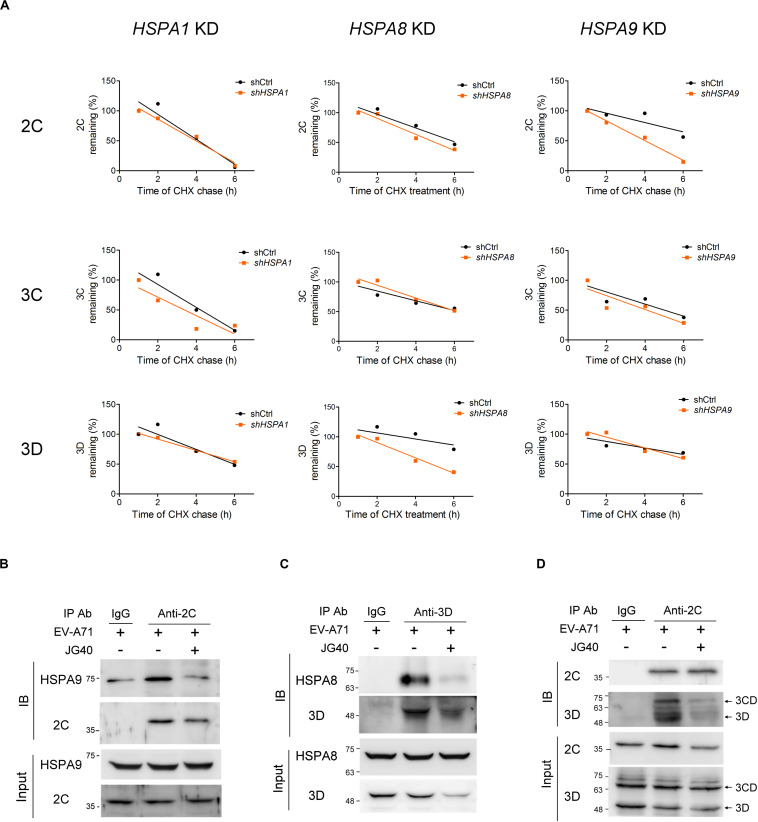
HSPA8 and HSPA9 stabilize viral proteins to facilitate replication complex formation. **(A)** RD cells treated with shCtrl or with HSPA1, HSPA8, or HSPA9 depleted were infected with EV-A71 (MOI = 5) at time 0. CHX (100 μg/ml) was added to the cell culture 9 h.p.i., and the viral protein levels at 1, 2, 4, and 6 h after CHX treatment were examined by immunoblot analysis. The stability levels of the viral proteins 2C, 3C, and 3D in the *HSPA1*-, *HSPA8-* and *HSPA9*-knockdown cells were compared to those in the shCtrl cells, as shown in the regression curves, and the immunoblot data for these regression curves are presented in Supplementary Figure S3. To perform the coimmunoprecipitation experiments, RD cells were infected with EV-A71 (MOI = 30), and JG40 was added to the cell culture 2 h.p.i. Cell lysates were harvested 5 h.p.i. and immunoprecipitated with the indicated antibodies or control IgG, and then, an immunoblot analysis was performed using the antibodies indicated. **(B)** The antibody against 2C coimmunoprecipitated HSPA9 with 2C, an outcome attenuated by JG40 treatment. **(C)** The antibody against 3D coimmunoprecipitated HSPA8 with 3D, an outcome also greatly reduced by JG40 treatment. **(D)** The antibody against 2C coimmunoprecipitated viral 3CD and 3D with 2C, a finding indicative of the formation of the replication complex. The replication complex formation was greatly disrupted by JG40 treatment.

Next, the stability levels of the viral 2C, 3C, and 3D proteins were examined in RD cells depleted of a specific HSP70 isoform to determine the HSP70 isomer that specifically regulated a viral protein. The results revealed that 2C stability was significantly reduced in the *HSPA9*-knockdown cells and that 3D stability was reduced in the *HSPA8*-knockdown cells. However, the stability of the 3C protein was not impacted in any of these three *HSP70*-knockdown cells (Figure 6A and Supplementary Figure S3). The substrate-chaperone relationship was further confirmed by coimmunoprecipitation experiments, which demonstrated that 2C interacted with HSPA9 (Figure 6B) and that 3D interacted with HSPA8 (Figure 6C). Notably, the interaction of viral proteins with respective HSP70s was greatly reduced in the presence of JG40. We also used coimmunoprecipitation experiments to reveal the impact of HSP70 inhibition on the formation of the replication complex. As shown in Figure 6D, the viral 2C protein formed a complex with the 3D and 3CD proteins in the EV-A71-infected cells; however, these interactions were significantly attenuated by the HSP70 inhibitor. The results indicate that different HSP70 chaperones are involved with the folding of different viral proteins during EV-A71 infection, which ultimately facilitates the formation of the replication complex.

### HSP70 Chaperone Proteins Participate in Viral Assembly and Release Processes

**FIGURE 7:**
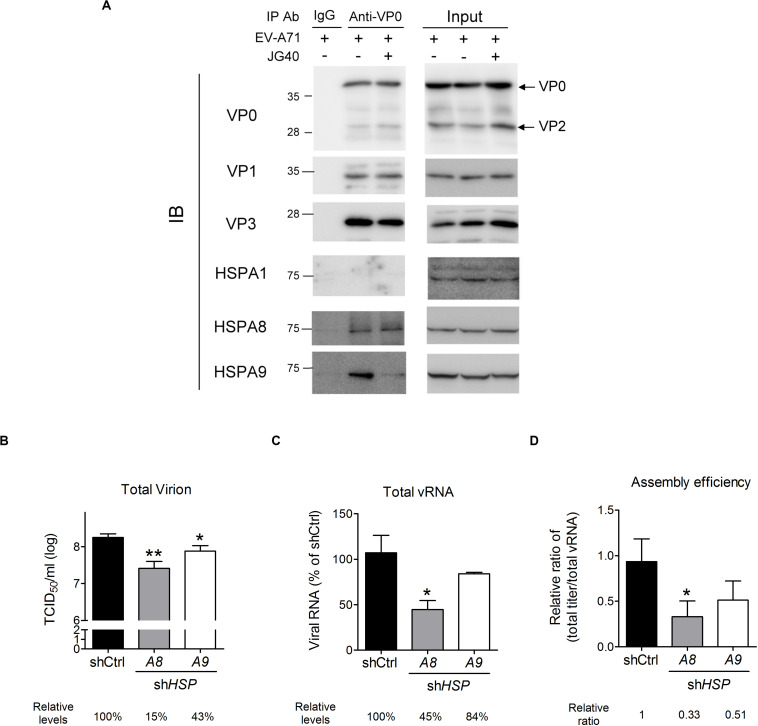
HSPA8 and HSPA9 are involved in EV-A71 viral particle assembly. **(A)** A coimmunoprecipitation experiment was performed to examine the effects of JG40 on viral capsid formation. RD cells were infected with EV-A71 (MOI = 1), and JG40 was added to the cell culture 8∼12 h.p.i. Cell lysates were harvested 12 h.p.i. and immunoprecipitated with anti-VP0 antibody or control IgG, followed by immunoblot analysis using the antibodies indicated. **(B–D)** RD cells depleted of HSPA8 or HSPA9 were infected with EV-A71 (MOI = 1). Twelve hours after infection, **(B)** the total viral titer in each group was determined by TCID_50_ assay, and **(C)** the level of total vRNA, prepared by collecting cultured supernatant and cell lysates, was determined by RT-qPCR analysis. The relative levels of virus or vRNA among groups are indicated by the number shown at the bottom, with that of the control knockdown cells set at 100%. **(D)** The relative ratios of total viral levels/total vRNA levels, representing the assembly efficiency, are shown. One-way ANOVA followed by Dunnett’s *post hoc* analysis was performed to compare the differences between the shCtrl cells and the *HSP70* knockdown cells. *P < 0.05 and **P < 0.01.

As shown in Figure 2C, JG40 treatment 8∼12 h.p.i. reduced viral particle production, as repeatedly observed in our experiments, albeit the decrease was only by 1.6–2-fold. To determine whether HSP70s might be involved in viral particle assembly, we first investigated whether HSP70s, acting as cochaperones for HSP90 (Geller et al., 2007), facilitated the protein folding of the P1 precursor and thus favored 3CD cleavage to generate VP0, VP1, and VP3 proteins. JG40 or 17-allylamino-17-demethoxygeldanamycin (17-AAG), an HSP90 inhibitor, was added to the cell culture 1 h.p.i., the cells were harvested at 4 h.p.i., and the processing of the P1 protein was examined. The immunoblot analysis demonstrated that, although 17-AAG significantly impaired P1 protein processing as anticipated, JG40 did not impact P1 processing (Supplementary Figure S4A). Next, we sought to determine whether HSP70s impacted viral particle encapsidation and maturation. The interaction between the viral 2C and VP3 proteins, required for viral encapsidation (Liu et al., 2010), was examined by coimmunoprecipitation experiments (Supplementary Figure S4B), whereas the maturation of the VP0 protein into the VP2 protein upon vRNA encapsidation was examined in the 160S infectious viral particles obtained *via* sucrose gradient fractionation (Supplementary Figure S4C). However, neither encapsidation nor maturation were impacted by JG40 treatment. The possibility that HSP70 inhibition might reduce viral capsid protein (VP0, VP1, and/or VP3) stability was also eliminated by the results of the CHX chase experiments (Supplementary Figures S4D–F). Finally, we performed coimmunoprecipitation experiments using an anti-VP0 antibody to investigate whether JG40 treatment administered 8∼12 h.p.i. might impact the interaction among viral capsid proteins and thus inhibit the formation of subviral or viral particles and whether HSP70s might be associated with viral capsid proteins. The results, as shown in Figure 7A, revealed that VP0 formed complexes with VP1 and VP3 efficiently in the presence or absence of JG40. HSPA8, and HSPA9, but not HSPA1, were found to be associated with capsid proteins. The interaction of HSPA9 with capsid proteins was significantly abrogated by JG40 treatment but that of HSPA8 was not. These results suggested that JG40 treatment administered 8∼12 h.p.i. did not impair, at least, the formation of protomers. Notably, JG40 treatment at this stage did not alter viral protein levels (Figure 7A, comparing the input protein levels in the groups with or without JG40) but did reduce total viral titers (Figure 2C). Thus, we speculated that HSP70 inhibition at this stage might affect the assembly of protomers into provirions and that HSPA8 and HSPA9 were likely involved in the processes.

To ascertain whether HSPA8 or HSPA9 was involved in viral particle assembly, RD cells depleted of HSPA8 or HSPA9 were infected with EV-A71, and the total viral titers were measured 12 h.p.i. The *HSPA8-* and *HSPA9-*knockdown cells produced only 15 and 43%, respectively, of the viral particles produced in the knockdown control cells (Figure 7B). However, since HSPA8 and HSPA9 also impact viral entry, translation and replication, the reduction in total vRNA (cell-associated + extracellular) could be expected (Figure 7C) and indicated a reduction in viral levels. To determine the effects of *HSP70* knockdown exclusively on viral particle assembly, we considered the rates by which vRNA was converted into viral particles to represent assembly efficiency, which was normalized to reflect the total viral titers to the abundance of total vRNA (Figure 7D). The resulting ratios indicated that the cells depleted of HSPA8 or HSPA9 showed reduced assembly efficiency, although for the HSPA9-depleted cells the reduction did not reach the level of significance (Figure 7D). The results suggest that HSPA8, and likely HSPA9, are also involved in viral assembly processes.

**FIGURE 8:**
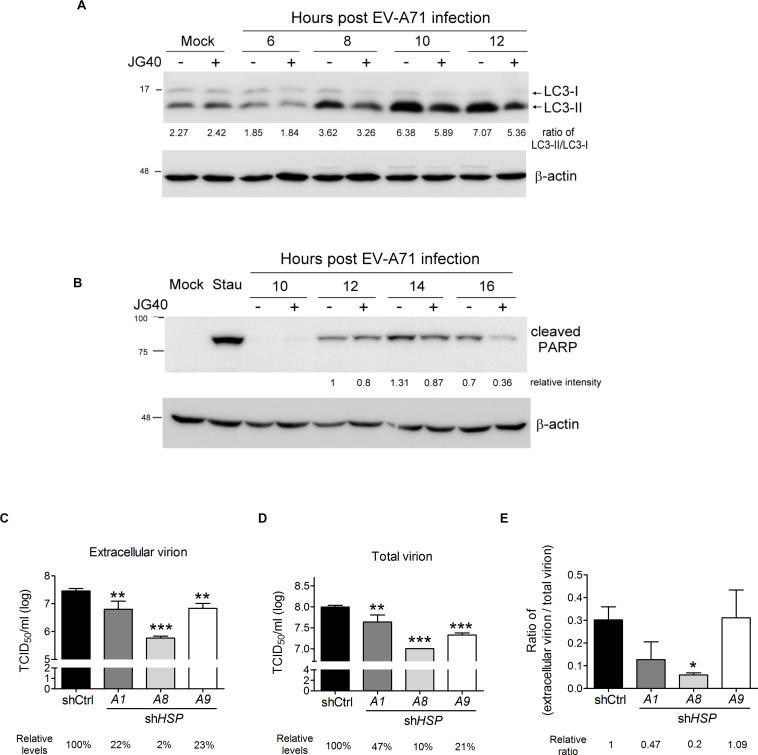
HSP70 inhibition suppresses autophagy- and apoptosis-mediated viral release. RD cells were infected with EV-A71 (MOI = 10), and JG40 was added to the cell culture 2 h.p.i. **(A)** Cells were harvested for immunoblotting and detection of LC3 at 6, 8, 10 and 12 h.p.i. The ratios of LC3-II/LC3-I, indicative of autophagy activation, are shown by the numbers below the blot. **(B)** Cells were collected for immunoblotting and assessed for cleaved PARP, indicative of apoptosis, 10, 12, 14, and 16 h.p.i. The relative intensities of the protein bands are shown by the numbers below the blot, based on the absence of JG40 at 12 h.p.i. arbitrarily set at 1. Staurosporine (0.1 μM) incubated with cells for 16 h was used as a positive control for the apoptosis assay. **(C–E)** RD cells depleted of HSPA1, HSPA8, or HSPA9 were infected with EV-A71 (MOI = 1). Twelve hours postinfection, the levels of extracellular virion **(C)** and total virion **(D)** were determined by TCID_50_ assays. The relative levels of viral titers among groups are indicated by the number shown at the bottom, with that of the control knockdown cells set at 100%. **(E)** The relative ratios of extracellular viral titer/total viral titer, representing the release efficiency, were calculated with that of the control knockdown cells set at 1. One-way ANOVA followed by Dunnett’s *post hoc* analysis was performed to compare differences between the shCtrl cells and the *HSP70*-knockdown cells. *P < 0.05, **P < 0.01, and ***P < 0.001.

Enterovirus A71 can be released from cells in a lytic or non-lytic manner. The former mechanism is the result of cellular apoptosis (Ventoso et al., 1998; Cong et al., 2016; Li et al., 2017), and the latter is dependent on secretory autophagy (Bird et al., 2014; Robinson et al., 2014; Chen et al., 2015). Therefore, we examined whether HSP70 inhibition would impact EV-A71-induced autophagy or apoptosis. JG40 was added to the EV-A71-infected cells 2 h.p.i. and the cells were harvested at different time points after infection. The immunoblots showing LC3 and cleaved PARP were assessed to determine the effects of JG40 on autophagy and apoptosis, respectively. As shown in Figure 8A, viral infection-induced autophagy, indicated by the conversion of LC3-I to LC3-II, appeared 8 h.p.i. and was maintained to 12 h.p.i. However, the autophagy level was reduced in the presence of JG40. As shown in Figure 8B, viral infection induced apoptosis starting 12 h.p.i. and peaking 14 h.p.i., in parallel with the time of cell lysis. Similarly, the apoptosis rate was also significantly reduced in the presence of JG40. To further identify which HSP70s were involved in viral release processes, RD cells depleted of a specific HSP70 were infected with EV-A71, and extracellular viral titers (Figure 8C) and total viral titers (Figure 8D) were determined 15 h.p.i., the time at which approximately 30–40% of the infected cells were lysed. Similarly, the ratio of extracellular viral titer/total viral titer was used to represent the release efficiency. The resulting ratios indicated that cells depleted of HSPA1 or HSPA8 had lower release efficiency (Figure 8E), and especially HSPA8, suggesting that HSPA1 and HSPA8 are involved in viral release processes.

### JG40 Inhibits the Replication of Divergent Enteroviruses

**FIGURE 9:**
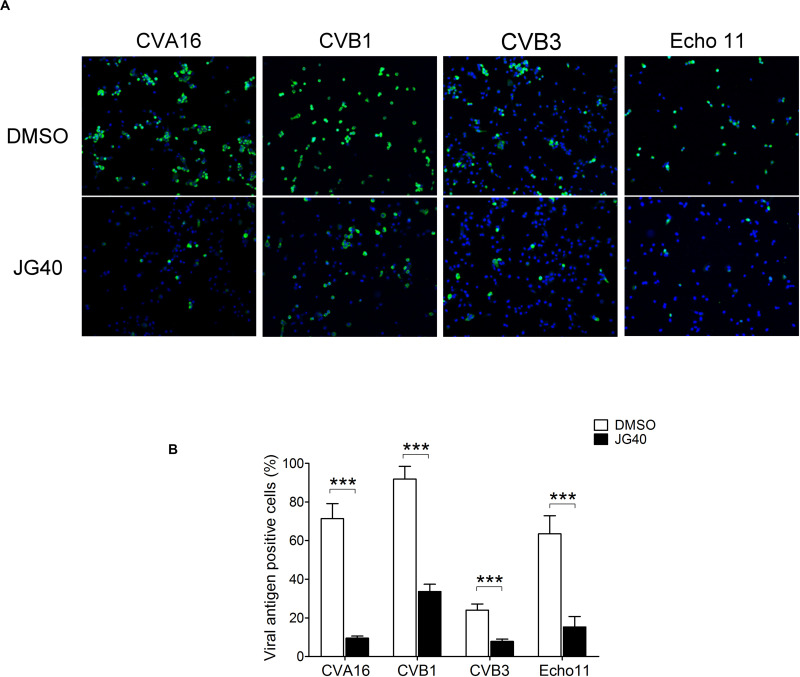
HSP70 inhibition suppresses the replication of various enteroviruses. **(A)** RD cells were treated with DMSO or JG40 1 h prior to infection with various EVs as indicated (MOI = 1). The drug remained in the medium throughout the experiments. Nine hour postinfection, the cells were fixed, permeabilized and stained for viral capsid antigens using specific antibodies. A representative image of each virus-infected culture is shown. **(B)** Viral antigen-positive cells were counted in at least 10 fields at 100× magnification. The average % of viral antigen (+) cells was defined by the number of viral antigen (+) cells divided by the number of DAPI (+) cells. Two-tailed Student’s *t*-test was performed to compared the differences between cells infected with each virus with or without JG40 treatment. ***P < 0.001.

Since the replication mechanisms of EVs are highly conserved, we speculated that HSP70s could also impact the life cycles of other EVs. To test this hypothesis, JG40 was applied to cell cultures infected with divergent EVs, namely, CVA16, CVB1, CVB3, and echovirus (Echo) 11. The expression of viral antigens was detected by immunofluorescent staining using specific antibodies against viral capsid proteins 9 h.p.i. (Figure 9A). The quantitative results revealed that, albeit with different infection rates, the replication of all the viruses tested was significantly inhibited by JG40 treatment (Figure 9B), findings indicative of the importance of HSP70s in replication cycles of all the EVs.

## Discussion

During viral infection, a large quantity of viral proteins are rapidly synthesized, whereby protein folding may become a limiting step. Therefore, most viruses exploit cellular chaperone proteins not only to facilitate the folding of their own proteins but also for the regulation of cellular processes to create a favorable environment for their proliferation. The HSP70 network chaperones constitute a large protein family that plays important roles in a variety of cellular processes (Bukau et al., 2000; Hartl and Hayer-Hartl, 2002; Finka et al., 2015). These proteins are also known to participate in the replication cycles of many viruses (Mayer, 2005). In the current study, we concurrently investigated the roles of subnetwork HSP70 chaperone proteins, HSPA1, HSPA8, and HSPA9, in EV-A71 proliferation and systematically dissected their roles in each step of the EV-A71 replication cycle. Our results revealed that the HSP70 chaperones are involved in all phases of the EV-A71 life cycle, including viral entry, translation, replication, assembly and release, and that each step requires multiple HSP70 chaperone proteins. It is believed that the HSP70 chaperones regulate these steps through their roles in folding newly synthesized proteins, promoting the assembly or disassembly of oligomeric structures and controlling the activity and stability levels of regulatory proteins (Bukau et al., 2000; Hartl and Hayer-Hartl, 2002; Finka et al., 2015).

The requirement for HSPA1, HSPA8, and HSPA9 in both viral entry and translation processes is intriguing. In investigating the entry step, we intentionally added CHX to prevent vRNA translation. Thus, the viral protein expression level is not a confounding factor here. We found that the depletion of any of these three chaperones significantly reduced viral internalization, suggesting their non-redundant roles in the cellular processes that regulate viral entry. EV-A71 is known to be internalized *via* SCARB2-mediated endocytosis, which is dependent on clathrin and dynamin 2 (Hussain et al., 2011; Lin et al., 2012). HSPA8 is a well-characterized chaperone protein that dissociates the clathrin triskelion from clathrin-coated vesicles and is essential for receptor-mediated endocytosis (Chappell et al., 1986). Thus, it is not surprising to that depleting HSPA8 had an impact on viral entry into the cells. HSPA1 is the major stress-inducible chaperone protein in cells. However, it is also expressed at basal levels under normal conditions and is thus possibly involved in the entry step. HSPA1 has been reported to facilitate the formation of the replication complex for HCV (Chen et al., 2010), and its role in endocytosis had not been reported. One study reported that HSPA1 forms a complex with clathrin and FKBP36, a testes-specific peptidyl-prolyl *cis/trans* isomerase, and proposed a possible role for this complex in clathrin uncoating, specifically in spermatocytes. However, no further experiments were conducted in this study (Jarczowski et al., 2008). HSPA9, also known as mortalin, is a housekeeping mitochondrial chaperone that participates in the folding and import of mitochondrial proteins through the mitochondrial membrane into the mitochondrial lumen (Dahlseid et al., 1994). However, HSPA9 is also found in multiple subcellular localizations, such as the ER and Golgi, cytoplasmic vesicles, and at the cell surface (Ran et al., 2000). HSPA9 is involved in tumor processes (Flachbartova and Kovacech, 2013) and in heparan sulfate proteoglycan (HSPG)-mediated endocytosis (Wittrup et al., 2010). However, few reports have described roles for HSPA9 in the virus replication cycle. Therefore, the mechanisms by which HSPA1 and HSPA9 regulate cellular processes that promote EV-A71 entry into cells require further investigation.

IRES-mediated translation requires not only canonical translational factors but also many ITAFs (Lozano and Martinez-Salas, 2015). Many of these ITAFs originally found in the nucleus are translocated to the cytoplasm during EV infection (Lloyd, 2015). In our previous studies, we demonstrated that EV-A71 2A^pro^ and 3C^pro^ proteases enhance IRES translational activity (Su et al., 2018), with 2A^pro^ being an essential protease for the production of the truncated eIF4G required for IRES-dependent translation and may also indirectly enhance translation by cleaving nucleoporin and disrupting the nuclear pore to facilitate ITAF translocation (Park et al., 2010; Watters and Palmenberg, 2011), and 3C^pro^ may disrupt stress granules by cleaving G3BP and promoting vRNA translation after eIF2α phosphorylation (White et al., 2007). It has been envisioned that HSP70 network chaperones enhance IRES activity through diverse mechanisms. First, the chaperone proteins may regulate folding and enhance the stability of viral proteins 2A^pro^ and 3C^pro^ to augment viral translation. Regulation of 3C stability by HSP70 is shown in Figure 5C. This study did not investigate the effects of JG40 on 2A^pro^ protein stability because a 2A^pro^-specific antibody was unavailable. However, our unpublished data showing overexpressed Flag-tagged 2A in the presence or absence of JG40 supported the notion that JG40 reduced Flag-2A stability (data not shown). Second, HSP70s may facilitate the disassembly of the nuclear ribonucleoprotein complex to release ITAFs. Third, HSP70s may participate in the transport of ITAFs across the nuclear pore. Fourth, HSP70s may have roles in the assembly of ITAFs as a translation-competent ribonucleoprotein complex in the cytoplasm. Fifth, as has been well known, some translational factors essential for IRES activity are derived from the cleaved products of original proteins, such as truncated eIF4G (Lamphear et al., 1993), eIF5B (White et al., 2011) and far upstream element binding protein 2 (FBP2) (Chen et al., 2013), and HSP70 chaperone activity may be required for the folding of these newly cleaved polypeptides. Given the many potential cellular processes that are capable of regulating IRES activity, it is expected that many HSP70s will be found to be involved in EV-A71 IRES activity.

We demonstrated that HSP70 inhibition significantly decreased the stability of EV-A71 proteins 2C, 3C, and 3D in virus-infected cells (Figure 5), leading to the reduced formation of the replication complex (Figure 6D). The results highlight the importance of HSP70s at the active replication phase. Interestingly, the folding of different viral proteins was governed by different HSP70s, indicating that viruses efficiently exploit cellular chaperones simultaneously to promote viral protein folding. While we could clearly demonstrate the substrate-chaperone relationship between 2C and HSPA9 and between 3D and HSPA8, the chaperone critical for 3C stability was not identified through our knockdown strategy. We speculate that 3C protein folding is either regulated by isoforms of HSP70 that were not included in this study or is regulated by multiple HSP70s redundantly. In either case, depletion of one particular HSP70 would not have abrogated 3C stability, whereas JG40 treatment, which inhibits all HSP70s, significantly reduced 3C stability. The mechanism by which HSP70s regulate 3C stability awaits elucidation.

The formation of EV-A71 viral progeny is a highly complex, stepwise process. Our results demonstrated that HSP70 inhibition at the late stage of the viral life cycle, i.e., 8∼12 h.p.i., led to reduced levels of viral particles, albeit the reduction was only 1.6–2-fold (Figure 2C). However, at this stage, the reduction phenomenon was not caused by lower levels of viral proteins due to JG40 treatment (Figure 7A, which shows a comparison of the total input proteins) but a role for HSP70s is suggested for viral particle assembly. Our data excluded the possibility that JG40 treatment influenced the cleavage of the P1 precursor protein or the VP0 protein, and it did not impact the interactions among VP0, VP1, and VP3 or between VP3 and 2C, suggesting that HSP70s might regulate a later stage of the assembly processes, for example, the oligomerization of protomers to form subviral particles or provirions. HSPA8 and HSPA9 were speculated to be involved because they are both associated with viral capsid proteins. In support of this notion, the viral particle assembly efficiency in the *HSPA8-* or *HSPA9-*knockdown cells was greatly reduced. Given that HSPA9 governed the stability of 2C protein (Figure 6A), an important factor for viral encapsidation, it is reasonable to suggest that the assembly efficiency in the *HSPA9*-knockdown cells was decreased because of reduced amount of 2C protein. HSPA8 is important for viral particle assembly, but the mechanism is far less clear because the association of HSPA8 with viral capsid proteins was insensitive to JG40 treatment (Figure 7A). The results may partly explain why JG40 treatment 8∼12 h.p.i., which blocks chaperone activity, led to a reduction in virion formation of only 1.6–2-fold. We speculated that HSPA8 may not directly chaperone the capsid proteins, but may facilitate viral particle formation by associating capsid proteins with other proteins essential for particle assembly, which does not involve chaperone activity. Alternatively, it is possible that the HSPA8 chaperone function required for viral particle assembly, if the chaperone activity indeed plays a role, may have been executed before JG40 treatment 8 h.p.i. The underlying mechanisms for HSPA8 regulation of EV-A71 viral particle assembly await further investigation.

Enterovirus A71 infection triggers the activation of autophagy and apoptosis in cells, which ultimately leads to viral particle release. In this study, we found HSPA1 and HSPA8 participating in viral particle release. It was previously reported that overexpressing *HSPA1* in peritoneal mesothelial cells exposed to lipopolysaccharide (LPS) could enhance autophagy, which is dependent on c-Jun N-terminal kinase (JNK) activation, and thus conferred protection to the cells against LPS-induced apoptosis (Li et al., 2011). EV-A71 infection also upregulates HSPA1 expression at the late stage of the virus replication cycle (our unpublished data). Thus, autophagy is expected to be augmented, thereby facilitating viral release. HSPA8 is known to be a key component in chaperone-mediated autophagy (CMA), in which HSPA8 recognizes proteins containing a KFERQ motif sequence and directly targets them for translocation to the lumen of lysosomes for degradation (Agarraberes and Dice, 2001). However, EV-A71 capsid proteins do not contain the KFERQ motif sequence, and viral release is not thought to be realized through the lysosomal degradation pathway. Interestingly, one recent paper demonstrated that the CMA pathway mediates the degradation of Unc-51 like autophagy activating kinase 1 (ULK1), which not only plays a key role in the initiation of autophagy but also regulates the autophagosome-lysosome fusion mediated by syntaxin 17 (STX17) (Wang et al., 2018). ULK1 physically interacts with STX17, and the interaction enhances the fusion of autophagosomes with lysosomes, whereas PKCα phosphorylates ULK1 and decreases the interaction between ULK1 and STX17, which prevents autolysosome formation. Interestingly, phosphorylation of ULK1 enhances its interaction with HSPA8 and increases its degradation through the CMA pathway. PKCs are serine/threonine protein kinases that can be activated by increased concentrations of diacylglycerol or calcium ions (Ca^2^^+^) (Nishizuka, 1995). Increased levels of cytosolic Ca^2^^+^ have been observed in the PV-infected cells (Irurzun et al., 1995). Together, these results reveal a mutual regulatory mechanism involving canonical autophagy and CMA and highlight an important role for HSPA8 in viral release; that is, HSPA8 recruits ULK1 and induces its degradation *via* CMA during EV-A71 infection, thus decreasing autolysosome formation and increasing secretory autophagic release, which is in line with the mechanism by which EVs employ 3C^pro^ to cleave the SNARE complexes to prevent the fusion of autophagosome with lysosome (Corona et al., 2018; Mohamud et al., 2018).

Collectively, the data from this study demonstrate that several HSP70 chaperones are required for the EV-A71 life cycle. Among the three HSP70s studied here, HSPA8 and HSPA9 seem particularly important. Although EV-A71 infection may induce abundant stress-inducible chaperones to benefit viral replication, the results presented here indicate that constitutively expressed HSP70s have non-replaceable roles in the viral life cycle. This study did not investigate the roles of HSPA5, an ER-resident chaperone, in the EV-A71 replication cycle because of the poor efficiency and off-target effects of the shRNA clones available to study it. However, a role for HSPA5 in EV-A71 replication should not be ruled out. It was recently shown that EV-A71 infection induces the redistribution of HSPA5 to the cytosol, thereby facilitating virus replication (Jheng et al., 2016). In summary, this study reveals that EV-A71 exploits multiple HSP70 chaperone proteins at each step of its replication cycle and that a single HSP70 often functions at multiple viral infection stages. Given the important functions of HSP70s in multiple steps of most of the EV replication cycle, targeting HSP70s is a promising broad-spectrum antiviral strategy.

## Data Availability Statement

The original contributions presented in the study are included in the article/Supplementary Material, further inquiries can be directed to the corresponding author.

## Author Contributions

L-HH and C-JC conceived the project. L-HH wrote the manuscript. Y-SS, P-YH, J-SL, and Y-HP conducted the experiments. All authors contributed to the article and approved the submitted version.

## Conflict of Interest

The authors declare that the research was conducted in the absence of any commercial or financial relationships that could be construed as a potential conflict of interest.
