# Supplementary material for: The Heat Shock Protein 70 Family of Chaperones Regulates All Phases of the Enterovirus A71 Life Cycle
Source: Front Microbiol. 2020 Jul 14;11:1656. doi: 10.3389/fmicb.2020.01656 (PMC7371988; doi:10.3389/fmicb.2020.01656)
Supplement: Supplementary file 1 [file Data_Sheet_1.PDF]

SUPPLEMENTARY MATERIALS

Supplementary Materials and Methods

Viral Particle Separation by Sucrose Gradient Ultracentrifugation

RD cells infected with EV-A71 (MOI=1) were treated with DMSO or JG40 (5 μM) 8~12 h.p.i. The virus-infected cultured supernatant and cell lysates were harvested at 12 h.p.i. by freeze-thawing the cells twice. Cell debris was removed by using a 0.45 μm filter. The virus supernatant was concentrated through a 15% sucrose cushion by ultracentrifugation using a Beckman SW41 Ti rotor (37,000 rpm for 2.5 h at 4°C). Virus pellets were resuspended in 1 ml purification buffer (10 mM Tris-HCl, pH 7.5, 200 mM NaCl and 50 mM $MgCl_2$) and further purified by a 15%-35%-45% (2 ml-4 ml-4 ml) discontinuous sucrose gradient ultracentrifugation at 32,000 rpm for 120 min at 4°C using a Beckman SW41 Ti rotor. After centrifugation, fractions were collected from the top of tube in 0.5 ml aliquots. Five microliters of each fraction were used for immunoblot analysis using anti-VP0/VP2 antibody.

Supplementary Table S1: Primer sequences used for PCR

Gene	Primer sequences
HSPA1A/A1B	Forward: 5' gATCAACgACggAgACAAgC 3'<br>Reverse: 5' gCTgCgAgTCgTTgAAgTAg 3'
HSPA5	Forward: 5' gCTCgACTCgAATTCCAAAg 3'<br>Reverse: 5' TgACACCTCCCACAgTTTCA 3'
HSPA8	Forward: 5’ ggAggTggCACTTTTgATgT 3’<br>Reverse: 5’ AgCAgTACggAggCgTCTTA3’
HSPA9	Forward: 5' CTTgTTTCAAggCgggATTA 3'<br>Reverse: 5' CgCTCACCATCTgCTgTAAA 3'
EV-A71 3D	Forward: 5’gATCTTgTCgATggCCCTAA 3’<br>Reverse: 5' gATCTTgTCgATggCCCTAA 3'
Luc	Forward: 5’ CgTTATTTATCggAgTTgCAgTTg 3’<br>Reverse: 5’ AAATCCCTggTAATCCgTTTTAgA 3’
GAPDH	Forward: 5' gTATTgggCgCCTggTCACC 3'<br>Reverse: 5' CgCTCCTggAAgATggTgATgg 3'

Supplementary Figures

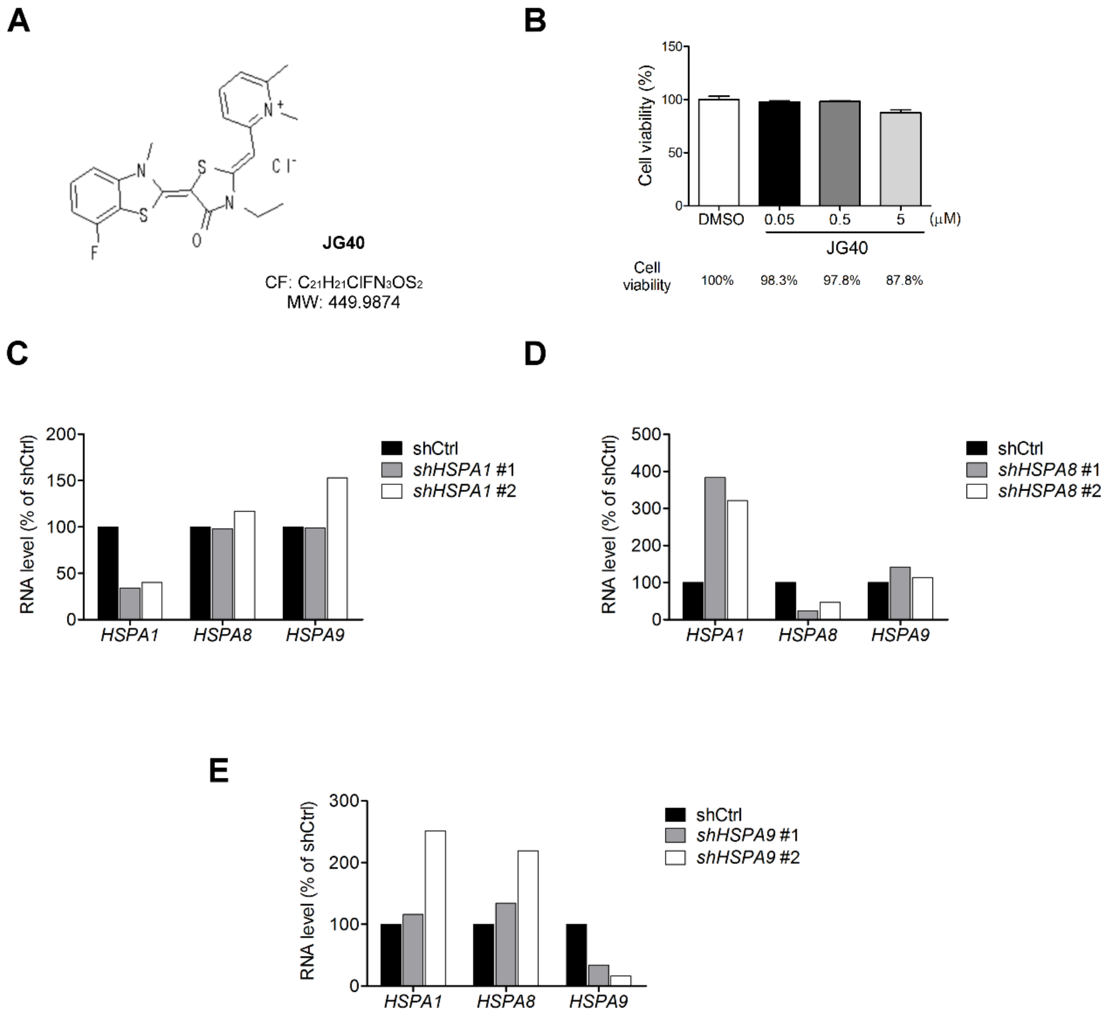

SUPPLEMENTARY FIGURE S1 **(A)** The chemical structure and information of JG40 are shown. (**B**) Cellular toxicity of JG40 was examined by measuring the viability of RD cells treated with the drug (0.05, 0.5 or 5 μM) for 13 h using the alamarBlue methods, with that of DMSO-treated cell set at 100%. (**C-E**) The off-target effects of the shRNA clones targeting *HSPA1* **(C)**, *HSPA8* **(D)** or *HSPA9* **(E)** were checked by RT-qPCR analysis to determine whether these shRNAs would downregulate the expression of other HSP70 isoforms in addition to the isoform targeted.

A

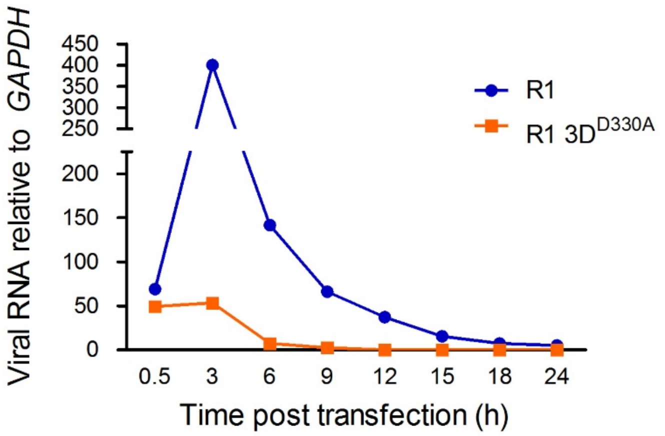

B

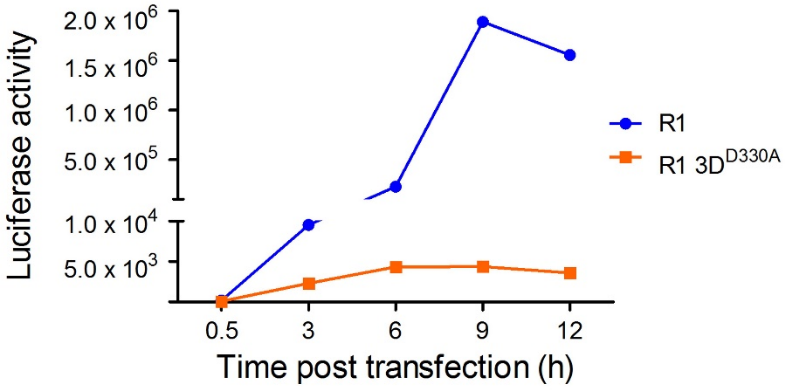

SUPPLEMENTARY FIGURE S2 The profiles of vRNA replication and luciferase activity in the RD cells transfected with the R1 or the R1 3D^{D330A} replicon RNA are shown. The replicon RNAs were transcribed *in vitro* from the replicon DNA templates and 1 µg of each of the replicon RNA was transfected into RD cells. The cells were harvested at the indicated time points post RNA transfection. Viral RNA levels were determined by RT-qPCR **(A)** and luciferase levels were measured by activity assays **(B)**.

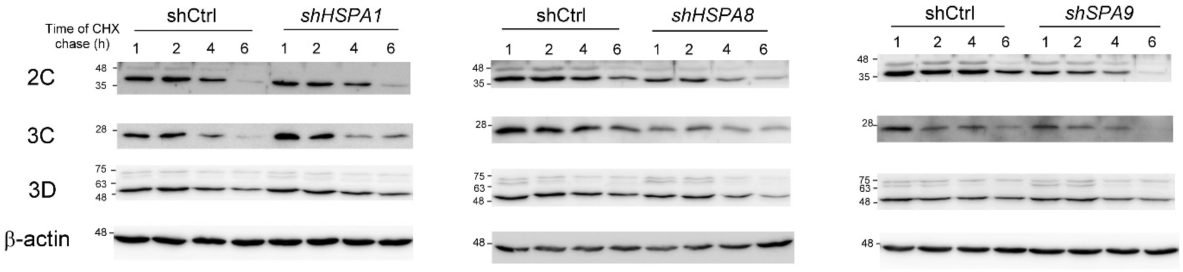

SUPPLEMENTARY FIGURE S3 The immunoblot images of viral proteins 2C, 3C and 3D for the CHX chase experiments in **FIGURE 6A** conducted in control knockdown or *HSPA1*, *HSPA8* and *HSPA9* knockdown cells.

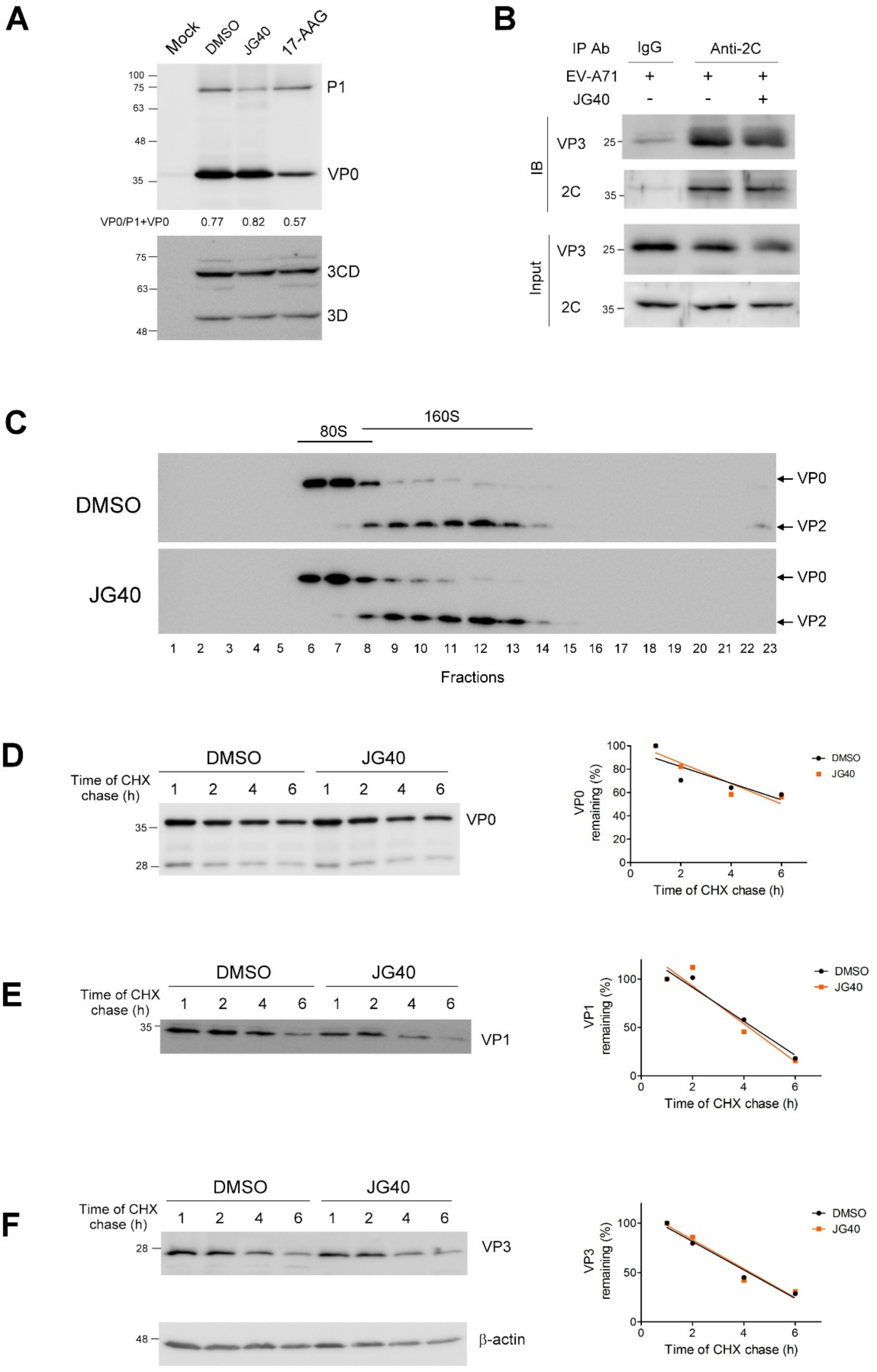

SUPPLEMENTARY FIGURE S4 HSP70 inhibition does not impact the cleavage of P1 precursor protein or VP0 protein, the interaction between VP3 and 2C, or the

stabilities of viral structural proteins. (**A**) RD cells were infected with EV-A71 (MOI=30) at time 0. JG40 (5 μM) or 17-AAG (0.3 μM) was added to cell culture 1 h.p.i. and cells were harvested at 4 h.p.i for immunoblot analysis using anti-VP0 antibody (upper panel) or anti-3D antibody (lower panel). The cleavage efficiency of P1 precursor protein was defined by the ratio of VP0/VP0+P1, indicated by the numbers shown at the bottom. (**B**) RD cells were infected with EV-A71 (MOI=30) and JG40 was added to cell culture at 2 h.p.i. Cell lysates were harvested 5 h.p.i. for co-immunoprecipitation experiment using anti-VP0 antibody, followed by immunoblot analysis with anti-VP3 or anti-2C antibody. (**C**) The 160S infectious and the 80S empty viral particles were separated by ultracentrifugation through a 15%-35%-45% discontinuous sucrose gradient. Immunoblotting of each fraction was performed using anti-VP0/VP2 antibody. The fractions containing mainly the VP0 protein correspond to the 80S empty particles, whereas the fractions containing mainly the VP2 protein correspond to the 160S infectious viral particles. The effects of JG40 on the stability of structural proteins VP0 (**D**), VP1 (**E**) or VP3 (**F**) were determined by CHX chase experiment as described in the legend to **Figure 5**.
